# 6-(Adamantan-1-yl)-3-(3-fluoro­phen­yl)-1,2,4-triazolo[3,4-*b*][1,3,4]thia­diazole

**DOI:** 10.1107/S1600536810046428

**Published:** 2010-11-17

**Authors:** Mahmood-ul-Hassan Khan, Shahid Hameed, Tashfeen Akhtar, Helen Stoeckli-Evans

**Affiliations:** a Department of Chemistry, Quaid-i-Azam University, Islamabad 45320, Pakistan; b Institute of Physics, University of Neuchâtel, rue Emile-Argand 11, CH-2009 Neuchâtel, Switzerland

## Abstract

The title mol­ecule, C_19_H_19_FN_4_S, displays *C*_s_ mol­ecular symmetry, in which the crystallographic mirror plane bis­ects the adamantan-1-yl unit while the 3-fluoro­phenyl triazole ring is located on the mirror plane. The F atom of the 3-fluoro­phenyl ring is positionally disordered [occupancy ratio 0.9:0.1]. In the crystal, π–π inter­actions between the triazole and phenyl rings occur [centroid–centroid distance = 3.5849 (7) Å] and weak C—H⋯F inter­actions form a ribbon propagating in [010].

## Related literature

For the biological significance of fused heterocycles, see: Khan *et al.* (2010*a* ▶,*b* ▶); Demirbas *et al.* (2005 ▶); Amir *et al.* (2007 ▶); Ashok *et al.* (2007 ▶); Palekar *et al.* (2009 ▶); Serwar *et al.* (2009 ▶); Akhtar *et al.* (2007 ▶, 2008*a* ▶,*b* ▶). For the activity of adamantyl derivatives, see: Kadi *et al.* (2007 ▶); Kouatly *et al.* (2009 ▶); Zahid *et al.* (2009 ▶). For a related structure, see: Khan *et al.* (2009 ▶). For standard bond lengths, see: Allen *et al.* (1987 ▶).
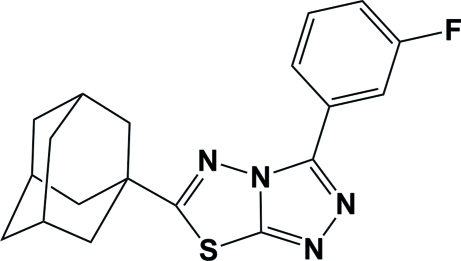


## Experimental

### 

#### Crystal data

C_19_H_19_FN_4_S
*M* *_r_* = 354.44
Monoclinic, 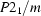

*a* = 11.6385 (16) Å
*b* = 6.6555 (5) Å
*c* = 11.6634 (16) Å
β = 117.379 (14)°
*V* = 802.25 (17) Å^3^
*Z* = 2
Mo *K*α radiation
μ = 0.22 mm^−1^
*T* = 173 K
0.34 × 0.30 × 0.11 mm

#### Data collection

Stoe IPDS-2 diffractometer
6164 measured reflections
1708 independent reflections
1223 reflections with *I* > 2σ(*I*)
*R*_int_ = 0.031

#### Refinement

*R*[*F* ^2^ > 2σ(*F* ^2^)] = 0.030
*wR*(*F* ^2^) = 0.074
*S* = 0.95
1708 reflections
154 parameters
4 restraints
H atoms treated by a mixture of independent and constrained refinement
Δρ_max_ = 0.20 e Å^−3^
Δρ_min_ = −0.26 e Å^−3^

### 

Data collection: *X-AREA* (Stoe & Cie, 2006 ▶); cell refinement: *X-AREA*; data reduction: *X-RED32* (Stoe & Cie, 2006 ▶); program(s) used to solve structure: *SHELXS97* (Sheldrick, 2008 ▶); program(s) used to refine structure: *SHELXL97* (Sheldrick, 2008 ▶); molecular graphics: *PLATON* (Spek, 2009 ▶) and *Mercury* (Macrae *et al.*, 2006 ▶); software used to prepare material for publication: *SHELXL97* (Sheldrick, 2008 ▶), *PLATON* and *publCIF* (Westrip, 2010 ▶).

## Supplementary Material

Crystal structure: contains datablocks I, global. DOI: 10.1107/S1600536810046428/kp2286sup1.cif

Structure factors: contains datablocks I. DOI: 10.1107/S1600536810046428/kp2286Isup2.hkl

Additional supplementary materials:  crystallographic information; 3D view; checkCIF report

## Figures and Tables

**Table 1:** Hydrogen-bond geometry (Å, °)

*D*—H⋯*A*	*D*—H	H⋯*A*	*D*⋯*A*	*D*—H⋯*A*
C11—H11*A*⋯F1*A*^i^	0.99	2.67	3.526 (3)	145

Symmetry code: (i) 
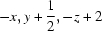
.

**Fig. 1.**
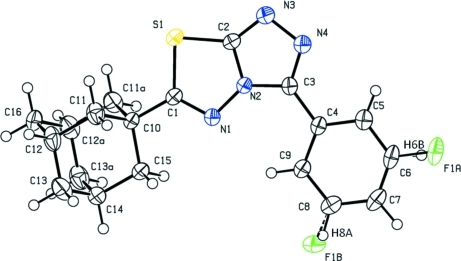
A view of the molecular structure of the title molecule with the displacement ellipsoids drawn at the 50% probability level [symmetry code (a) = x, 3/2 - y, z; The positional disorder of the 3-fluorophenyl group is also illustrated: atoms F1A and H8A (occupancy 0.45) and atoms F1B and H6B (occupancy 0.05)].

**Fig. 2.**
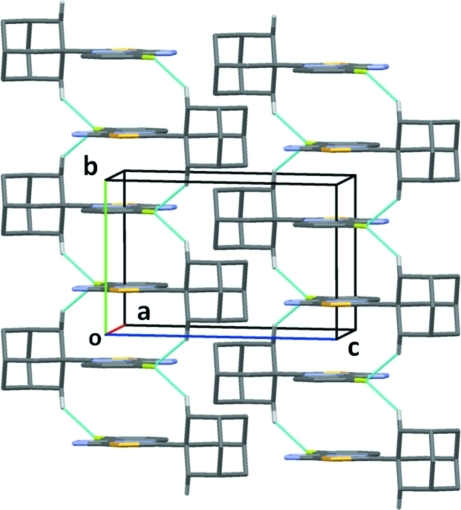
The crystal packing of the title compound along the a axis. The C—H···F interactions are shown as dashed cyan lines.

## supplementary crystallographic information

### Comment

Fused heterocycles have received widespread attention in the recent years due to their outstanding biological applications [Khan *et al.* 2010*a*; Demirbas *et al.*, 2005; Amir *et al.*, 2007; Ashok *et al.*, 2007; Palekar *et al.*, 2009]. Moreover, compounds bearing an adamantyl moiety are gaining prominence due to their biological and pharmaceutical potential [Kadi *et al.*, 2007; Kouatly *et al.*, 2009; Zahid *et al.*, 2009]. The title compound was synthesised in continuation of our previous studies on the synthesis and biological screening of five membered heterocycles [Akhtar *et al.*, 2007, 2008*a*, 2008*b*; Serwar *et al.*,2009; Khan *et al.*, 2009, 2010*b*].

Bond distances (Allen *et al.*, 1987) and bond angles are normal and similar to those reported for the 2-fluorophenyl derivative (Khan *et al.*, 2009). The 3-fluorophenyl moiety is positionally disordered, with atom F1 having occupancies of 0.45/0.05 (F1A/F1B), where F1A is bonded to atom C6, while atom F1B is bonded to atom C8 (Fig. 1).

The title molecule possesses crystallographic mirror symmetry; the mirror plane bisects the adamantane moiety while the rest of the molecule lies in the mirror plane. The 3-fluorophenyl moiety is positionally disordered. The 2-fluorophenyl derivative, however, crystallised in the orthorhombic space group Pbca and the molecule possess no crystallographic symmetry. The 2-fluorophenyl ring is inclined to the triazole ring by 48.6 (1)°, to eliviate steric hindrance.

In the crystal of the title compound there are weak C—H···F interactions [C11—H11A···F1A^i^, H···F 2.67 Å, C—H···F 145 °, symmetry code (i) -*x*, *y* + 1/2, -*z* + 2)] leading to the formation of a one-dimensional ribbon-like structure running in [010] (Fig. 2). There are also π–π interactions with the centroid-to centroid distance between the mean planes of the triazole ring (N2,C2,N3,N4,C3) and symmetry related phenyl rings (C4—C9) being 3.5849 (7) Å [the perpendicular distance is 3.3278 (1) Å with a slippage of 1.333 Å].

### Experimental

A mixture of 4-amino-5-(3-fluorophenyl)-2*H*-1,2,4-triazole-3(4*H*)-thione (0.2 g, 0.56 mmol) and adamantane-1-carboxylic acid (0.10 g, 0.56 mmol) was refluxed in the presence of POCl_3_ (5.0 mL) for 4 h [Khan *et al.* 2010*a*]. The reaction mixture was cooled to RT, poured into crushed ice and neutralized using solid potassium carbonate until p_H_ 8. The precipitated solid was filtered off, washed with excess water and recrystallised from chloroform to give colourless plate-like crystals, suitable for X-ray analysis.

### Refinement

A region of electron denstiy was located near atom C8 indicating a positional disorder of the 3-fluorophenyl moiety. In the final cycles of least-squares refinement atom F1 was split to give F1a (occupancy 0.45) bonded to atom C6 and atom F1b (occupancy 0.05) bonded to atom C8. Bond length C8—F1b was refined with a distance restraint of 1.36 (2) Å. H-atoms H6a, H8b and H7 were refined with distance restraints of 0.95 (2) A% with *U*_iso_(H) = 1.2*U*_eq_(C). The remaining H-atoms were included in calculated positions and treated as riding atoms: C—H = 0.95, 0.99, and 1.00 Å for CH-aromatic, CH_2_ and CH-methine H-atoms, respectively, with *U*_iso_(H) = 1.2*U*_eq_(parent C-atom).

### Crystal data

**Table:**

C_19_H_19_FN_4_S	*F*(000) = 372
*M**_r_* = 354.44	*D*_x_ = 1.467 Mg m^−^^3^
Monoclinic, *P*2_1_/*m*	Mo *K*α radiation, λ = 0.71073 Å
Hall symbol: -P 2yb	Cell parameters from 4261 reflections
*a* = 11.6385 (16) Å	θ = 3.1–26.0°
*b* = 6.6555 (5) Å	µ = 0.22 mm^−^^1^
*c* = 11.6634 (16) Å	*T* = 173 K
β = 117.379 (14)°	Plate, colourless
*V* = 802.25 (17) Å^3^	0.34 × 0.30 × 0.11 mm
*Z* = 2	

### Data collection

**Table:**

Stoe IPDS-2 diffractometer	1223 reflections with *I* > 2σ(*I*)
Radiation source: fine-focus sealed tube	*R*_int_ = 0.031
graphite	θ_max_ = 26.1°, θ_min_ = 3.4°
φ and ω scans	*h* = −14→14
6164 measured reflections	*k* = −7→8
1708 independent reflections	*l* = −14→14

### Refinement

**Table:**

Refinement on *F*^2^	Primary atom site location: structure-invariant direct methods
Least-squares matrix: full	Secondary atom site location: difference Fourier map
*R*[*F*^2^ > 2σ(*F*^2^)] = 0.030	Hydrogen site location: inferred from neighbouring sites
*wR*(*F*^2^) = 0.074	H atoms treated by a mixture of independent and constrained refinement
*S* = 0.95	*w* = 1/[σ^2^(*F*_o_^2^) + (0.0449*P*)^2^] where *P* = (*F*_o_^2^ + 2*F*_c_^2^)/3
1708 reflections	(Δ/σ)_max_ = 0.001
154 parameters	Δρ_max_ = 0.20 e Å^−^^3^
4 restraints	Δρ_min_ = −0.26 e Å^−^^3^

### Special details

**Table:**

Geometry. Bond distances, angles *etc*. have been calculated using the rounded fractional coordinates. All su's are estimated from the variances of the (full) variance-covariance matrix. The cell e.s.d.'s are taken into account in the estimation of distances, angles and torsion angles
Refinement. Refinement of *F*^2^ against ALL reflections. The weighted *R*-factor *wR* and goodness of fit *S* are based on *F*^2^, conventional *R*-factors *R* are based on *F*, with *F* set to zero for negative *F*^2^. The threshold expression of *F*^2^ > σ(*F*^2^) is used only for calculating *R*-factors(gt) *etc*. and is not relevant to the choice of reflections for refinement. *R*-factors based on *F*^2^ are statistically about twice as large as those based on *F*, and *R*- factors based on ALL data will be even larger.

### Fractional atomic coordinates and isotropic or equivalent isotropic displacement parameters (Å^2^)

**Table:**

	*x*	*y*	*z*	*U*_iso_*/*U*_eq_	Occ. (<1)
S1	0.42509 (5)	0.75000	0.98501 (5)	0.0307 (2)	
F1A	−0.2195 (2)	0.75000	1.1429 (2)	0.0500 (6)	0.900
N1	0.17319 (15)	0.75000	0.85412 (15)	0.0251 (5)	
N2	0.20898 (15)	0.75000	0.98446 (16)	0.0243 (5)	
N3	0.35605 (17)	0.75000	1.18934 (17)	0.0325 (6)	
N4	0.23242 (17)	0.75000	1.18047 (16)	0.0311 (6)	
C1	0.27709 (18)	0.75000	0.84027 (19)	0.0249 (6)	
C2	0.33636 (19)	0.75000	1.06956 (19)	0.0268 (6)	
C3	0.14499 (19)	0.75000	1.05806 (19)	0.0257 (6)	
C4	0.00535 (19)	0.75000	1.0101 (2)	0.0248 (6)	
C5	−0.0419 (2)	0.75000	1.1000 (2)	0.0318 (7)	
C6	−0.1732 (2)	0.75000	1.0556 (2)	0.0342 (7)	
C7	−0.2599 (2)	0.75000	0.9272 (2)	0.0378 (8)	
C8	−0.2117 (2)	0.75000	0.8390 (2)	0.0367 (8)	
C9	−0.08020 (19)	0.75000	0.8790 (2)	0.0299 (7)	
C10	0.27522 (18)	0.75000	0.71113 (18)	0.0254 (6)	
C11	0.34448 (16)	0.9374 (3)	0.69694 (15)	0.0362 (5)	
C12	0.34071 (17)	0.9366 (3)	0.56371 (15)	0.0413 (6)	
C13	0.20148 (17)	0.9360 (3)	0.45854 (15)	0.0428 (6)	
C14	0.1337 (2)	0.75000	0.4720 (2)	0.0382 (8)	
C15	0.1355 (2)	0.75000	0.6040 (2)	0.0368 (7)	
C16	0.4093 (2)	0.75000	0.5501 (2)	0.0441 (9)	
F1B	−0.2859 (18)	0.75000	0.7166 (11)	0.052 (5)	0.100
H7	−0.3504 (11)	0.75000	0.902 (2)	0.0450*	
H8A	−0.276 (4)	0.75000	0.7516 (18)	0.0440*	0.900
H9	−0.04830	0.75000	0.81730	0.0360*	
H11A	0.30140	1.05980	0.70660	0.0440*	
H11B	0.43540	0.93830	0.76530	0.0440*	
H12	0.38550	1.05930	0.55460	0.0490*	
H5	0.01550	0.75000	1.19000	0.0380*	
H13B	0.19930	0.93710	0.37270	0.0510*	
H14	0.04200	0.75000	0.40260	0.0460*	
H15A	0.08990	0.87050	0.61220	0.0440*	0.500
H15B	0.08990	0.62950	0.61220	0.0440*	0.500
H16A	0.40810	0.75000	0.46460	0.0530*	
H16B	0.50060	0.75000	0.61760	0.0530*	
H13A	0.15650	1.05780	0.46590	0.0510*	
H6B	−0.21 (3)	0.75000	1.11 (2)	0.0410*	0.100

### Atomic displacement parameters (Å^2^)

**Table:**

	*U*^11^	*U*^22^	*U*^33^	*U*^12^	*U*^13^	*U*^23^
S1	0.0210 (3)	0.0411 (4)	0.0298 (3)	0.0000	0.0116 (2)	0.0000
F1A	0.0514 (10)	0.0583 (11)	0.0639 (12)	0.0000	0.0467 (9)	0.0000
N1	0.0251 (8)	0.0263 (10)	0.0252 (8)	0.0000	0.0128 (7)	0.0000
N2	0.0218 (8)	0.0275 (9)	0.0245 (8)	0.0000	0.0115 (6)	0.0000
N3	0.0268 (9)	0.0403 (12)	0.0291 (9)	0.0000	0.0118 (7)	0.0000
N4	0.0285 (9)	0.0371 (12)	0.0285 (9)	0.0000	0.0139 (7)	0.0000
C1	0.0219 (9)	0.0230 (11)	0.0301 (10)	0.0000	0.0123 (8)	0.0000
C2	0.0207 (9)	0.0285 (12)	0.0289 (10)	0.0000	0.0095 (8)	0.0000
C3	0.0283 (10)	0.0249 (12)	0.0269 (10)	0.0000	0.0153 (9)	0.0000
C4	0.0260 (10)	0.0206 (11)	0.0320 (10)	0.0000	0.0169 (9)	0.0000
C5	0.0344 (12)	0.0298 (13)	0.0361 (11)	0.0000	0.0205 (9)	0.0000
C6	0.0368 (12)	0.0282 (12)	0.0520 (14)	0.0000	0.0329 (11)	0.0000
C7	0.0270 (11)	0.0337 (14)	0.0581 (15)	0.0000	0.0243 (11)	0.0000
C8	0.0254 (11)	0.0401 (15)	0.0414 (13)	0.0000	0.0126 (10)	0.0000
C9	0.0256 (10)	0.0336 (13)	0.0326 (11)	0.0000	0.0153 (9)	0.0000
C10	0.0217 (10)	0.0288 (12)	0.0276 (10)	0.0000	0.0131 (8)	0.0000
C11	0.0436 (9)	0.0339 (10)	0.0359 (8)	−0.0108 (7)	0.0223 (7)	−0.0049 (7)
C12	0.0535 (10)	0.0393 (10)	0.0405 (9)	−0.0168 (8)	0.0298 (8)	−0.0033 (7)
C13	0.0565 (11)	0.0416 (11)	0.0355 (8)	0.0138 (8)	0.0257 (8)	0.0081 (7)
C14	0.0291 (11)	0.0576 (17)	0.0279 (11)	0.0000	0.0132 (9)	0.0000
C15	0.0232 (10)	0.0586 (17)	0.0290 (10)	0.0000	0.0123 (9)	0.0000
C16	0.0313 (12)	0.073 (2)	0.0357 (12)	0.0000	0.0220 (10)	0.0000
F1B	0.030 (7)	0.084 (12)	0.031 (7)	0.0000	0.006 (7)	0.0000

### Geometric parameters (Å, °)

**Table:**

S1—C1	1.773 (2)	C10—C15	1.529 (3)
S1—C2	1.725 (2)	C11—C12	1.534 (2)
F1A—C6	1.353 (3)	C12—C13	1.517 (3)
F1B—C8	1.285 (12)	C12—C16	1.524 (3)
N1—N2	1.379 (2)	C13—C14	1.514 (3)
N1—C1	1.292 (3)	C14—C15	1.530 (3)
N2—C3	1.371 (3)	C5—H5	0.9500
N2—C2	1.353 (3)	C6—H6B	0.9 (3)
N3—N4	1.394 (3)	C7—H7	0.955 (17)
N3—C2	1.308 (3)	C8—H8A	0.95 (2)
N4—C3	1.318 (3)	C9—H9	0.9500
C1—C10	1.496 (3)	C11—H11A	0.9900
C3—C4	1.455 (3)	C11—H11B	0.9900
C4—C9	1.389 (3)	C12—H12	1.0000
C4—C5	1.390 (3)	C13—H13A	0.9900
C5—C6	1.370 (4)	C13—H13B	0.9900
C6—C7	1.368 (3)	C14—H14	1.0000
C7—C8	1.380 (3)	C15—H15A	0.9900
C8—C9	1.380 (4)	C15—H15B	0.9900
C10—C11	1.535 (2)	C16—H16A	0.9900
C10—C11^i^	1.535 (2)	C16—H16B	0.9900
			
C1—S1—C2	88.25 (11)	C13—C14—C13^i^	109.66 (19)
N2—N1—C1	108.20 (17)	C13^i^—C14—C15	109.73 (12)
N1—N2—C2	118.84 (18)	C10—C15—C14	109.9 (2)
N1—N2—C3	135.60 (19)	C12—C16—C12^i^	109.20 (19)
C2—N2—C3	105.56 (17)	C4—C5—H5	121.00
N4—N3—C2	104.65 (18)	C6—C5—H5	121.00
N3—N4—C3	109.67 (18)	C5—C6—H6B	122.00
S1—C1—N1	115.88 (15)	C7—C6—H6B	115.00
S1—C1—C10	121.10 (17)	C6—C7—H7	119.4 (13)
N1—C1—C10	123.02 (19)	C8—C7—H7	122.7 (13)
S1—C2—N2	108.83 (15)	C7—C8—H8A	114 (3)
S1—C2—N3	138.93 (19)	C9—C8—H8A	125 (3)
N2—C2—N3	112.2 (2)	C4—C9—H9	120.00
N2—C3—N4	107.9 (2)	C8—C9—H9	120.00
N2—C3—C4	126.26 (18)	C10—C11—H11A	110.00
N4—C3—C4	125.9 (2)	C10—C11—H11B	110.00
C3—C4—C5	117.99 (19)	C12—C11—H11A	110.00
C3—C4—C9	122.1 (2)	C12—C11—H11B	110.00
C5—C4—C9	119.9 (2)	H11A—C11—H11B	108.00
C4—C5—C6	118.3 (2)	C11—C12—H12	109.00
F1A—C6—C5	118.5 (2)	C13—C12—H12	109.00
F1A—C6—C7	118.4 (2)	C16—C12—H12	109.00
C5—C6—C7	123.2 (2)	C12—C13—H13A	110.00
C6—C7—C8	117.9 (2)	C12—C13—H13B	110.00
C7—C8—C9	121.1 (2)	C14—C13—H13A	110.00
F1B—C8—C7	122.2 (10)	C14—C13—H13B	110.00
F1B—C8—C9	116.8 (10)	H13A—C13—H13B	108.00
C4—C9—C8	119.6 (2)	C13—C14—H14	109.00
C1—C10—C11	110.08 (11)	C15—C14—H14	109.00
C1—C10—C15	109.90 (19)	C13^i^—C14—H14	109.00
C1—C10—C11^i^	110.08 (11)	C10—C15—H15A	110.00
C11—C10—C15	109.02 (11)	C10—C15—H15B	110.00
C11—C10—C11^i^	108.71 (18)	C14—C15—H15A	110.00
C11^i^—C10—C15	109.02 (11)	C14—C15—H15B	110.00
C10—C11—C12	109.55 (15)	H15A—C15—H15B	108.00
C11—C12—C13	109.96 (17)	C12—C16—H16A	110.00
C11—C12—C16	109.64 (15)	C12—C16—H16B	110.00
C13—C12—C16	109.24 (16)	H16A—C16—H16B	108.00
C12—C13—C14	109.39 (16)	C12^i^—C16—H16A	110.00
C13—C14—C15	109.73 (12)	C12^i^—C16—H16B	110.00
			
C2—S1—C1—N1	0.00 (1)	N4—C3—C4—C5	0.00 (1)
C2—S1—C1—C10	180.00 (1)	N4—C3—C4—C9	180.00 (1)
C1—S1—C2—N2	0.00 (1)	C3—C4—C5—C6	180.00 (1)
C1—S1—C2—N3	−180.00 (1)	C9—C4—C5—C6	0.00 (1)
C1—N1—N2—C2	0.00 (1)	C3—C4—C9—C8	180.00 (1)
C1—N1—N2—C3	180.00 (1)	C5—C4—C9—C8	0.00 (1)
N2—N1—C1—S1	0.00 (1)	C4—C5—C6—F1A	180.00 (1)
N2—N1—C1—C10	180.00 (1)	C4—C5—C6—C7	0.00 (1)
N1—N2—C3—C4	0.00 (1)	F1A—C6—C7—C8	180.00 (1)
C3—N2—C2—N3	0.00 (1)	C5—C6—C7—C8	0.00 (1)
N1—N2—C2—S1	0.00 (1)	C6—C7—C8—C9	0.00 (1)
C3—N2—C2—S1	180.00 (1)	C7—C8—C9—C4	0.00 (1)
N1—N2—C3—N4	−180.00 (1)	C1—C10—C11—C12	−179.45 (16)
C2—N2—C3—C4	180.00 (1)	C15—C10—C11—C12	−58.8 (2)
C2—N2—C3—N4	0.00 (1)	C11^i^—C10—C11—C12	59.90 (19)
N1—N2—C2—N3	180.00 (1)	C1—C10—C15—C14	180.00 (1)
N4—N3—C2—S1	180.00 (1)	C11—C10—C15—C14	59.27 (12)
N4—N3—C2—N2	0.00 (1)	C10—C11—C12—C13	59.6 (2)
C2—N3—N4—C3	0.00 (1)	C10—C11—C12—C16	−60.5 (2)
N3—N4—C3—C4	−180.00 (1)	C11—C12—C13—C14	−60.1 (2)
N3—N4—C3—N2	0.00 (1)	C16—C12—C13—C14	60.2 (2)
N1—C1—C10—C11	120.09 (13)	C11—C12—C16—C12^i^	60.3 (2)
N1—C1—C10—C15	0.00 (1)	C13—C12—C16—C12^i^	−60.27 (19)
S1—C1—C10—C15	180.00 (1)	C12—C13—C14—C15	60.2 (2)
S1—C1—C10—C11	−59.91 (13)	C12—C13—C14—C13^i^	−60.4 (2)
N2—C3—C4—C5	180.00 (1)	C13—C14—C15—C10	−60.28 (14)
N2—C3—C4—C9	0.00 (1)		

Symmetry codes: (i) *x*, −*y*+3/2, *z*.

### Hydrogen-bond geometry (Å, °)

**Table:**

*D*—H···*A*	*D*—H	H···*A*	*D*···*A*	*D*—H···*A*
C11—H11A···F1A^ii^	0.99	2.67	3.526 (3)	145

Symmetry codes: (ii) −*x*, *y*+1/2, −*z*+2.
